# Some Effects of Inebriety on the Teeth and Jaws2Written for the Section on Stomatology of the American Medical Association.

**Published:** 1905-10

**Authors:** T. D. Crothers


### International Dental Journal.

Vol. XXVI. October, 1905. No. 10.

### Original Communications.[1]

### SOME EFFECTS OF INEBRIETY ON THE TEETH AND JAWS.[2]

BY T. D. CROTHERS, M.D.[3]

Some years ago, through the courtesy of Dr. Talbot, my attention was called to the degenerations of the teeth and jaws in persons who had used spirits and drugs to excess. General marks of degeneration seen in the head and face and other parts of the body have been commonly noted in inebriates, but we owe to Dr. Talbot the credit of showing that certain of these defects were traceable to spirit- and drug-taking both as an active and predisposing cause.

My work for nearly thirty years has been almost exclusively confined to the study of inebriates from spirit- and drug-taking. This has given me an opportunity to note many facts along these lines and confirm and verify the statements of others. In an analysis of the histories of a large number of inebriates, at least seventy per cent. will be found to inherit from their ancestors neurotic defects and faults of vitality and nutrition. The remain-

[1] The editor and publishers are not responsible for the views of authors of papers published in this department, nor for any claim to novelty, or otherwise, that may be made by them. No papers will be received for this department that have appeared in any other journal published in the country.

[2] Written for the Section on Stomatology of the American Medical Association.

[3] Superintendent of Walnut Lodge Hospital, Hartford, Conn.

ing thirty per cent. are traceable to injuries, diseases, bad nutrition, and environment. The first class show marks of this neurotic heredity in defects of the body and deficient vitality. The second class are more prominent in general symptoms of anæmia, starvation, and poisoning.

The brain, nervous system, and functional activities reflect these conditions in many ways. Thus in the inherited class the head, face, teeth, and maxillaries all show degrees of symmetry which point to irregular and retarded growths and faults of nutrition. In the acquired class the faults of nutrition with irregular control and feeble brain function and vigor are prominent.

The direct action of spirits on the mouth and teeth is due in some degree to the rapid water-absorbing qualities of alcohol. Alcohol on the surface absorbs the moisture so rapidly that it becomes an irritant, corrugating the tissue. In the mouth, it has the same effect, which is neutralized in a measure by the excessive salivary discharge.

It has been noted that spirit- and beer-drinkers who take small quantities of spirits, sipping them over long periods of time, have defective teeth and gums, while others who drink spirits with large quantities of water, swallowing them rapidly, do not suffer so much. Persons who use beer and spirits to gratify the sense of taste and enjoy the flavors always suffer from degenerations of the mucous membrane, gums, and teeth. Often such defects are unnoticed until the spirits are withdrawn and the anæsthetic condition which has existed passes away; then the exposed nerves and crumbling teeth call for help.

The use of alcohol produces vasomotor paralysis, by which the circulation of the blood is retarded and deranged. The nutrition of tissue and cell is lowered and impoverished. Added to this there is an increase of toxins with leukocytes and deficient power of elimination; hence both starvation and poisoning follow. The terminal arterioles are deranged and the cell and dentrites suffer from defective nutrition and the irritation from the toxins. I think that the same process takes place in the terminal nerves of the teeth and gums that is noted in the nerves of the extremities in neuritis. There is, without doubt, a very close association between neuritis and dental degeneration, and in my experience they frequently follow each other.

Spirit- and drug-takers who complain of so-called rheumatism, particularly pain, stiffness of the joints, and defective locomotion, have neuritis and toxæmic states affecting the cell and dentrites at the extremities. The crumbling teeth and shrunken gums is another symptom of what appears to be the same pathological process of acute nerve poisoning.

The toxin of syphilis shows itself often in the faulty growth and condition of the teeth and gums. Lead and other poisons are manifest in the same way. The profession is not aware that alcohol is a toxæmic in its effects on nutrition, and it is only recently that we are confronted with the evidence that alcohol is cumulative in its effects.

The steady use of spirits, even in small doses, such as wine at meals taken with the utmost regularity for a long time, is a source of degeneration that is as clearly traceable as that of syphilis. In physics the continual tapping by a hammer on the hardest steel will after a time break up its tenacity and cohesiveness, causing it to crumble like chalk. In like manner the continued use of small doses of spirits acting on the vasomotor centres, deranging circulation and nutriton, increasing the toxins, and diminishing the power of elimination, produces permanent erosions and degenerations of cell and tissues. Fibrinous deposits and sclerosis of the nerve and tissue are always present in such cases. Metabolism and vitality are slowly and surely destroyed, and this condition is transmitted to the next generation, appearing in convulsive degenerations, recurrent insanities, and morbid impulses for spirits and drugs.

So-called moderate drinkers, who imagine that their temperate use of spirits is a virtue, with increased enjoyment of life, in reality cripple and destroy the next generation by transmitting to them defects that are continuous evidence of the degeneration from the use of spirits.

Dentists as well as physicians are constantly confronted with these evidences of the failure of parents to transmit to the next generation normal vigor and vitality. If associated with the use of spirits, either in excess or moderation, there is hypernutrition, the person using foods in excess, the toxæmic conditions are very greatly increased, and this is seen in the teeth and mouth.

Formerly unthinking physicians prescribed alcohol and drugs as remedies which covered up this condition. Often persons who have local inflammation of the dental nerves find that spirits and drugs give temporary relief, and very soon continue their use and become habitués. The defects of the teeth become intensified, and finally they are removed as a remedy.

Many persons believe that the local inflammation of the gums and teeth was the active cause of the drug addiction. Cocaine-taking is frequently contracted by the use of this drug for the effects on the gums. Other drug addictions may spring from the same cause, but it is evident that the localized effect on the teeth and gums point to some grave condition affecting the entire body, and the spirit- and drug-taking are only culminations of degenerations beginning long before.

Morphia-takers have defective teeth and a general marasmic condition of the mouth. The continued anæsthesia of the sensory centres not only increases the degeneration, but covers up all evidences of it. In some of the opium addictions there is great hyperæsthesia of the gums and teeth; in others marked anæsthesia appears. In most of the persons I see who use this drug to excess the active degeneration goes on in the mouth without any recognition of it, but the moment the drug is removed the condition becomes intensified and very prominent. Occasionally teeth that are sound become the centre of extreme pain. The entire dental nerve and its branches seem to be in a condition of acute inflammation, and even after the teeth have been removed obstinate neuralgias continue. A sudden acute localized inflammation of the dental nerves should be regarded as neuritis with distinct causes, prominent of which are alcohol and narcotics, also lead and mercury. I have met a few persons whose morphinism developed an acute hyperæsthesia of the maxillary nerves. Every effort to remove the drug was followed by a return of the pain and suffering, until finally the case became chronic and all treatment was abandoned. I have seen two such cases who are comfortable while using morphia, but the removal brought on the most aggravating suffering. In one instance reported to me, where the morphia had been removed, a surgical operation was resorted to and the nerve severed, but the pain continued. The removal of the teeth does not always check the pain. The nerve becomes permanently disorganized.

It is an interesting question in the study of the causes to know how far alcohol or other narcotics have had primary or secondary effects on the teeth and gums. Where dental defects existed before drug-taking some constitutional condition existed, usually manifest in that way. Some very unusual cases have come under my observation where profound shocks and derangements of nutrition have resulted in inflammation and erosion of the teeth. The following is an example:

A business man, aged forty-five years, temperate, with no heredity, was made unconscious by a lightning stroke in his immediate vicinity. Invalidism without any distinct symptoms lasted for two months; then suddenly he began to use spirits to great excess, and came under my care. In a short time the craze disappeared entirely, and acute maxillary inflammation began, extending to every tooth of the mouth. There was no evidence of decay, only extreme hyperæsthesia of the gums and tenderness of the teeth. All local and general treatment failed, and he went under the care of a nerve specialist. A year later he returned as a morphia-taker. His teeth had all been removed, but the pain along the maxillary continued except when under the narcotism of morphia.

A second example was a similar temperate business man, in good health up to the death of his wife from an accident. A few weeks later acute inflammation of the teeth and gums, with great sensitiveness to heat and cold in the mouth, came on. Active treatment by a dentist, with the removal of the teeth, was followed by improvement. He then became an alcoholic inebriate. Under my care he was treated with electricity, and recovered both from degeneration of the mouth and the use of spirits.

There was probably in these cases some profound toxæmic condition associated with the psychical shocks in both cases which localized in the maxillaries. I have the histories of several persons who before all use of spirits and drugs had a prolonged period of dental troubles in which nearly every tooth was filled, and later they all broke down. The history of specific taints with salivation from mercury and loss of teeth, than spirit- and drug-taking, are very common. In some persons forms of neurotic exhaustion precede the breaking down of the teeth; then comes spirit- and drug-taking as a local and general remedy.

A very interesting question is frequently asked concerning the effects of tobacco on the teeth and jaws. Evidently cigarette smoking is the most marked form of tobacco that appears to have its specific effect in the mouth, and persons who urge the antiseptic action of tobacco find it very difficult to substantiate their claims from any data known at present. Another interesting fact probably has some influence in this direction, although it cannot be very clearly made out. Thus the perversion and diminution or obliteration of the sense of taste follows the use of spirits. Such persons show abnormal impulses and crave for acids, sugars, salts, and irritant condiments, which influence the nerve terminals of the teeth and the mucous membrane of the mouth and its nutrition. The obliteration of the sense of smell is another factor encouraging localization of acute degeneration of the teeth and gums, or in the liver and kidneys.

I think that it can be clearly established that alcohol taken to great excess, either continuously or at intervals, has a profound influence on the teeth and gums. In paroxysms followed by a distinct free interval in which the anæsthetic effects pass away and a degree of restoration follows, the effects are less prominent. In the continuous use, as before mentioned, the nutrition and vigor of the teeth are always seriously impaired. Of course, there are certain distinct pathologic conditions which may concentrate and produce more serious effects in some particular organ of the body, and the teeth and gums may be one of these locations. The mucous membrane of the nose and throat is another. The liver and heart are also among the organs that register these special effects. Occasionally drug- and spirit-takers exhibit great fragility of the osseous system associated with degenerate teeth. This is probably owing to some faults in the diet, with absence of proper mineral matter and nutrition.

An observation made by the late Dr. Wright, some years ago, has been amply confirmed by my studies,—namely, that when syphilis and alcoholism are combined, degeneration of the teeth and maxillaries is a very common result. Both of these diseases are toxic and the teeth and jaws should be examined for evidence of their influence.

Another observation has been confirmed in many ways, and is this: In certain families, notably those who began with alcohol degeneracy, a marked persistence of diseases of the teeth and jaws is noted, extending through two or more generations and appearing in every member of the family. I have seen two incidents of this in which a great-grandfather, who was an inebriate, was followed by a numerous family, all of whom had defective and diseased teeth in early and middle life. This degeneration continued into the next generation, and was most marked. There were only three inebriates in a family of twenty-six, but every one had degenerate teeth.

I have been told that this form of degeneration is sometimes seen in syphilitic inheritance, and no doubt this is true.

I conclude these observations with a general summary: (1) Spirit- and drug-taking is a very potent cause of defective teeth and gums. It may be stated that a large percentage of degenerations of the mouth come directly or indirectly from this source. (2) Conditions of degeneration which localize in the teeth and gums may be the exciting causes of spirit- and drug-taking. The increasing prevalence and demand for narcotic drugs indicates low vitality of which the vigor of the teeth is a prominent part. (3) A study and correction of the injuries or beginnings of diseases in the mouth may be the removal and prevention of serious drug neurosis which may follow.

